# Public health round-up

**DOI:** 10.2471/BLT.16.010816

**Published:** 2016-08-01

**Do coffee and tea cause cancer?**

There is no conclusive evidence for a carcinogenic effect of drinking coffee or mate, a plant-based infusion. However, it is probable – although not certain – that these and other beverages cause oesophageal cancer when drunk very hot (above 65 degrees Celsius), according to scientists convened by the International Agency for Research on Cancer (IARC). Their conclusions, summarized in *The Lancet Oncology* in June, will be published as an IARC monograph. http://monographs.iarc.fr

**Figure:**
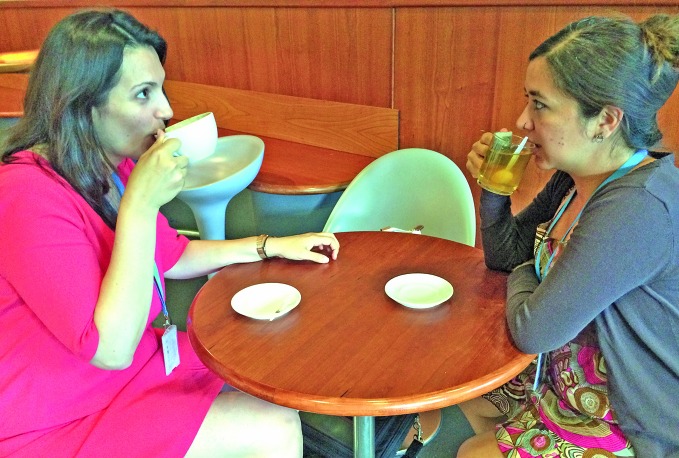
WHO/Fiona Fleck

## Global response to Zika 

Support for women and girls of child-bearing age is at the centre of a new revised plan that the World Health Organization (WHO) and its partners have issued to guide countries in their response to the Zika crisis. 

According to the revised plan, a key strategy for preventing the spread of the virus is ensuring that pregnant women, their partners and communities have the information they need to protect themselves from the virus.

 “This Zika virus strain and its complications represent a new type of public health threat which requires a unique and integrated strategy that places support for women and girls of child-bearing age at its core,” said WHO Director-General Dr Margaret Chan in the foreword of the revised plan.

The *Zika strategic response plan, July 2016 to December 2017* can be used by countries to guide their response to the Zika crisis. The plan revises the previous *Strategic response framework and joint operations plan, January–June 2016.*

Other elements of the plan include integrated vector management, sexual and reproductive health counselling and health education and care within the social and legal contexts of each country where Zika virus is being transmitted. About US$ 121.9 million is needed to implement the revised plan.

The growing Zika epidemic in the Americas and its link with birth defects and Guillain–Barré syndrome was declared a public health emergency in February. 

The virus is spreading increasingly to new areas where *Aedes aegypti*, the main type of mosquito that transmits the virus, are present.

Efforts to control these mosquitoes may slow the spread of the virus but cannot prevent it. However the development of a vaccine against Zika could take years. 

In the meantime, countries must prepare to manage the continued spread of the Zika virus and its consequences.

http://who.int/emergencies/zika-virus/response

## Zika and breastfeeding 

Mothers with suspected, probable or confirmed Zika virus infection should continue to breastfeed as there is currently no evidence that the virus can be transmitted in this way to their babies, according to updated WHO guidance.

The new guideline, released in June, can be used by governments, ministries of health, policy-makers and health-care workers for developing infant feeding policies and protocols on breastfeeding in areas where the Zika virus is circulating. 

The new guideline is based on a WHO-led systematic review of all the evidence on breastfeeding and Zika virus including the evidence from three mother–infant pairs. The guideline is an update of an interim guideline that was developed and released by WHO in February of this year. 

Thus, infants born to mothers with suspected, probable or confirmed Zika virus infection or who live in or have travelled to areas where Zika virus is circulating, should be fed according to standard infant feeding guidelines by WHO and the United Nations Children’s Fund. 

According to those guidelines, infants should start breastfeeding within one hour of birth, be exclusively breastfed for six months and have timely introduction of adequate, safe and properly-fed complementary foods, while continuing breastfeeding up to two years of age or beyond.

The Zika virus has been found in breast milk and therefore breast milk could be considered potentially infectious, but there are no documented reports to date of Zika virus being transmitted to infants through breastfeeding.

http://apps.who.int/iris/bitstream/10665/208875/1/9789241549660_eng.pdf

http://who.int/csr/resources/publications/zika/sexual-transmission-prevention

## HIV tests for infants 

WHO gave its prequalification stamp of approval in June to two innovative technologies for the diagnosis of HIV in children aged 18 months and younger. 

WHO prequalification gives United Nations agencies, donors and countries a guarantee of the tests’ quality, safety and performance, and therefore confidence that they can buy and use them effectively.

The two products: Alere™ q HIV-1/2 Detect, developed by Alere Technologies GmbH, and Xpert® HIV-1 Qual Assay, made by Cepheid AB, can be used to diagnose infants in as little as an hour, instead of sending a sample to a laboratory, which can take weeks or months to return a result.

These tests will allow many more infants to be diagnosed quickly and placed on life-saving treatment.

In 2015, out of more than 1.2 million infants born to HIV-positive mothers globally, just over half had access to an infant diagnostic test. 

That’s one of the reasons why only half of all children estimated to be living with HIV receive the treatment they need. 

The best way to diagnose HIV infection among infants is to use tests that look for evidence of the virus in the blood, rather than those that look for antibodies or antigens. 

The two diagnostic tests both use disposable cartridges which are pre-loaded with the chemicals needed to identify HIV in a blood sample. 

http://www.who.int/diagnostics_laboratory/cutting-edge-hiv-prequal

**Cover photo**

This month’s cover photo shows two sisters and their brother washing clothes in the river in Gao, the capital of the north-eastern Gao region of Mali. The family does not have access to a reliable water source near their home and so they have to walk 2 km to the river to do their laundry and to bathe.

**Figure:**
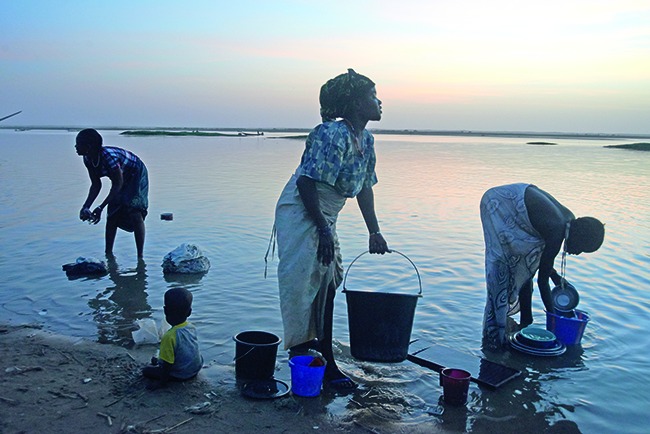
© UNICEF/NYHQ2014–0620/Phelps

## Medicines for Africa 

Officials from the European Union (EU) Commission, the African, Caribbean and Pacific Group of States and WHO met in Addis Ababa, Ethiopia in June with other partners to review the results of a programme to improve the quality and safety of medicines and make them more widely available.

The US$ 10 million Renewed Partnership programme that was launched in 2012 has been funded by the EU with the technical support of WHO. 

Many people in the African region struggle to get the medicines they need. Many of the medicines that are available are counterfeit or substandard products, due to weak regulatory control, while standard medicines are expensive and unaffordable. 

Moreover, few Africans are covered by social protection schemes and most people in the region must pay for medicines themselves, which can contribute to impoverishment. 

The goal of universal access to medicines for HIV has not been achieved and generic medicines for other conditions are available in only about 57% of health facilities across the region. 

The new programme has addressed many of these challenges. “The availability of quality medicines in health facilities is improving,” says Dr Matshidiso Moeti, WHO Regional Director for Africa. 

For example, the Ethiopian government has compiled a list of priority medicines to be covered by the national insurance scheme, marking a major step towards universal health coverage in that country, Moeti said. 

Pricing surveys conducted in Burundi and Mali have led to new legislation that serves to control prices in the private sector and, in turn, reduce out-of-pocket payments for patients. 

Regulators from most of the 15 countries that participated in the four-year programme took part in training workshops to acquire additional skills to assess and register medicines more efficiently.

In addition, Ethiopia and Mozambique are now screening more effectively for substandard and counterfeit products at ports of entry into the country, using improved detection techniques such as minilabs, Moeti told the meeting. 

National essential medicines lists in several of these countries have also been updated in line with the most recent evidence thus including more medicines for noncommunicable diseases and for children.

http://www.afro.who.int/en/clusters-a-programmes/hss/essential-medicines.html

## Pesticide residues in food 

New residue limits for more than 30 different pesticides in various foods have been adopted by the Codex Alimentarius Commission, the United Nations food standards body.

Pesticides are chemicals used to kill insects to prevent them from damaging crops. Pesticide residues often remain in food, such as grains, fruits and vegetables. 

To protect people’s health from high levels of such residues, the Joint Food and Agriculture Organization (FAO) and WHO Expert Meeting on Pesticide Residues, (JMPR), a group of independent international specialists, assessed the risks to human health posed by 30 pesticides.

Based on this health risk assessment and identification of safe exposure levels, the Codex Alimentarius Commission recommended specific limits for each pesticide-food combination at its 39th Session held in Rome at the end of June.

The Codex Alimentarius Commission, which is jointly run by the United Nations Food and Agriculture Organization and WHO, sets international food safety and quality standards to promote safer and more nutritious food for consumers worldwide. 

Codex standards serve in many cases as a basis for national legislation and provide the food safety benchmarks for international food trade.

Codex safety standards are based on a scientific assessment of all the available evidence. The JMPR is an international scientific expert group administered jointly by the FAO and WHO. The JMPR has met regularly since 1963 to review residues and analytical aspects of the pesticides, estimate the maximum residue levels, review toxicological data and estimate acceptable daily intakes for humans of the pesticides under consideration.

http://www.who.int/foodsafety/areas_work/food-standard/CAC

http://www.who.int/foodsafety/areas_work/chemical-risks/jmpr

**Looking ahead**

**28 July – World Hepatitis Day**

**19 September – United Nations Summit on Refugees and Migrants**

**14–20 November – World Antibiotic Awareness Week**

**1 December – World AIDS Day**

